# Bacterial agents and antibiotic resistance profiles of infections from different sites that occurred among patients at Debre Markos Referral Hospital, Ethiopia: a cross-sectional study

**DOI:** 10.1186/s13104-017-2584-y

**Published:** 2017-07-06

**Authors:** Wondemagegn Mulu, Bayeh Abera, Mulat Yimer, Tadesse Hailu, Haimanot Ayele, Dereje Abate

**Affiliations:** 1 Department of Medical Microbiology, Immunology and Parasitology, College of Medicine and Health Sciences, Bahir Dar University, P.O. Box 79, Bahir Dar, Ethiopia; 2 Department of Microbiology Laboratory, Debre Markos Referral Hospital, Debre Markos, Ethiopia

**Keywords:** Bacteria, Antibiotic resistance, Debre Markos Referral Hospital, Ethiopia

## Abstract

**Background:**

In developing countries like Ethiopia, infections with antibiotic resistant bacteria become a real threat. Hence, monitoring of local level antimicrobial resistance profile is indispensable to contain the spread of drug resistant bacteria and intervene poor awareness on antimicrobial resistance. Therefore, this study aimed at determining bacterial and antibiotic resistance profiles of infections from different sites that occurred among patients.

**Methods:**

Retrospective data recorded were analyzed on culture and drug susceptibility test results at Debre Markos Referral Hospital which were performed from 2011 to 2014. Drug susceptibility tests were performed using disk-diffusion technique. Chi square test was computed to compare the proportion of bacterial isolates with patients’ age and sex.

**Results:**

Out of 575 clinical samples processed, 280 (48.7%) were culture positive for aerobic bacteria pathogens. Wound 238 (41.4%) and urine 108 (18.8%) were the most frequent samples processed. Overall, *Staphylococcus aureus* (*S. aureus*) was the predominant isolate 100 (31.5%) followed by *Escherichia coli* (*E. coli*) *39* (13.8%), *Pseudomonas aeruginosa* (*P. aeruginosa*) 30 (10.3%) and *Salmonella* spp. 25 (8.9%). *P. aeruginosa* was the most frequent isolate followed by *S. aureus* from ear infection. *E. coli* was the leading isolate followed by *Klebsiella* spp. from urinary tract infection. *Salmonella* and *Shig*ella spp. were the most frequent isolates in stool in children below 5 years of age. *Neisseria gonorrhoeae* (*N. gonorrhoeae*) 16 (76.2%) was the most common isolate from urethral discharge. The overall multidrug-resistant Gram positive and Gram negative bacteria isolates were 113 (84.6%) and 96 (72.2%), respectively. Gram positive bacteria revealed resistance to cotrimoxazole (80%), gentamicin (83.1%), amoxicillin (85.1%), ampicillin (85.8%), penicillin (89.7%), clindamycin (93.2%) and erythromycin (90.9%). Gram negative bacteria showed resistance to cotrimoxazole (53.1%), amoxicillin (58.8%), ampicillin (70.4%), tetracycline (75.9%) and gentamicin (76.9%).

**Conclusions:**

Various bacterial infections linked with high levels of MDR bacteria pathogens are major health problems in the study area. Therefore, treatment of common bacterial infections in the study area needs to be guided by drug-susceptibility testing of isolates.

## Background

Bacterial infections caused by multidrug-resistant (MDR) bacteria are a growing threat worldwide [1]. They are major cause of morbidity and mortality in developing countries including Ethiopia. Antimicrobial resistance (AMR) is a major problem in both hospital and community acquired infections [2, 3].

The term MDR refers to a bacterium that is simultaneously resistant to a number of antimicrobial drugs belonging to different chemical classes or subclasses through various mechanisms [4]. Many bacteria species isolated from different clinical specimens have showed one or more resistance mechanisms to each of the major classes of antimicrobial agents [5, 6]. MDR bacteria acquire resistance by mutation or gene transfer through conjugation, transformation or transduction. These resistance mechanisms are widespread among common pathogens and cause considerable concern in several clinical situations in which treatment options have become very limited [5, 6].

AMR problem is challenging in low income countries due to high prevalence of infections, irrational uses of antimicrobials, over the counter availability of drugs and lack of clinical microbiology laboratories for antimicrobial susceptibility testing [3]. Infections caused by resistant bacteria adversely affect treatment outcomes, costs, disease spread and duration of illnesses, posing a serious challenge to the future chemotherapies [2, 7]. In addition to this, the battle between bacteria and their susceptibility to drugs is yet problematic among public, researchers, clinicians and drug companies who are looking for effective drugs [7].

The ongoing spread of resistant bacteria is partly due to the indiscriminate use of antibiotics. Antibiotic resistance emerges commonly when patients are treated with empiric antimicrobial drugs. This is critical in developing countries where the available antibiotics are misused [3, 6, 8]. To overcome these difficulties, monitoring of resistance profiles in the health institutions is needed [2, 6, 8, 9].

Majority of physicians and nurses in Ethiopia lack up to-date information on AMR [3]. Culture and drug susceptibility tests have been started since 2011 at Debre Markos Referral Hospital hence there was no documented comprehensive data on pathogenic bacteria and their drug susceptibility profiles from different sites of infections. Furthermore, ministry of health in its strategy includes monitoring of local antimicrobial resistance trends using standardized microbiological methods to intervene empirical therapy, poor awareness on AMR and to contain drug resistance. Therefore, the present study was conducted to determine bacteria and antibiotic resistance profiles of infections from different sites that occurred among patients at Debre Markos Referral Hospital, Ethiopia.

## Methods

### Study design, period and area

A Retrospective data review was made in January, 2015 on culture results of clinical specimens taken from different infection sites performed from September 2011 to December 2014 at Debre Markos Referral Hospital (DMRH). DMRH has more than 147 beds offering different specialized services. It receives patients from its catchment area and referrals from different areas of East Gojjam Zone. The hospital has four major wards namely, Internal Medicine, Surgery, Pediatrics and Gynecology and Obstetrics used for diagnosis and treatment of infected patients. Culture and drug susceptibility tests were conducted at DMRH microbiology laboratory for both out and inpatients. However, there is no laboratory facility for isolation of anaerobic bacteria from clinical specimens.

### Data collection

The age and sex of patients, the bacteria isolated and the drug susceptibility profiles were retrieved from DMRH microbiology, laboratory unit registration records using a standard data collection form. Laboratory records which had incomplete information of either age, sex or culture and drug susceptibility test results were excluded.

### Isolation and identification of bacteria

For the detection of pathogenic bacteria, all clinical samples were collected by standard microbiological technique [10]. The sources of specimens were pus/swab from wound, urine, ear discharge, blood, stool, urethral or cervical discharge, nasal or throat swab and CSF. Depending on the source of samples, each specimen were platted onto MacConkey agar, Blood agar, Mannitol Salt agar, Xylose lysine deoxycholate agar, Chocolate agar and Thayer–Martin agar (Oxoid, UK) and then incubated aerobically at 37 °C for 24 h. Bacterial species were identified as per the standard microbiological methods [10].

### Antimicrobial susceptibility testing

Antimicrobial susceptibility testing was done on Mueller–Hinton agar (Oxoid, England) using disk diffusion technique according to Kirby–Bauer method [11]. The antimicrobial agents tested were: ampicillin (10 µg), penicillin (10 IU), Oxacillin (1 µg), clindamycin (30 µg) amoxicillin (10 µg), ceftriaxone (30 µg), ciprofloxacin (5 µg), cloxacillin (5 µg), cotrimoxazole (25 µg), doxycycline (10 µg), tetracycline (30 µg), erythromycin (15 µg), chloramphenicol (30 µg), gentamicin (10 µg) (Oxoid, England). The antibiotic susceptibility profiles were interpreted based on Clinical and Laboratory Standards Institute (CLSI, 2006) guidelines [12]. Moreover, MDR profile was determined against different classes of antimicrobials: cephalosporin class (ceftriaxone), Aminoglycosides class (gentamycin); Fluoroquinolones class (ciprofloxacin, norfloxacin), Tetracycline class (doxycycline); Folate pathway inhibitors (cotrimoxazole); phenicols class (chloramphenicol); penicillin class (oxacillin, ampicillin, penicillin), Macrolides class (erythromycin) and Lincosamides class (clindamycin). Due to the varied definitions that are being used and differences in the antimicrobial agents that are used for routine antimicrobial susceptibility testing in clinical, referral and public health microbiology laboratories, no standard definitions for MDR have been agreed up on yet by the medical community. Therefore, we authors of this manuscript determined the MDR profiles by taking the literal definition of MDR as resistant to more than one antimicrobial agent [13].

#### Quality control

A standard bacteriological procedure was followed to maintain correct laboratory test results. American Type Culture collection (ATCC) standard reference strains *Escherichia coli* (*E. coli*) ATCC-25922, *Staphylococcus aureus* (*S. aureus*) ATCC 25923 and *Pseudomonas aeruginosa* (*P. aeruginosa*) ATCC 25853 were used to control quality of culture and drug susceptibility testing.

### Statistical analysis

Data were entered and analyzed using SPSS Statistical software Package (IBM Corp. Released 2011 IBM SPSS statistics for windows, version 20. Armonk, NY: IBM Corp). Chi square tests were computed to compare the proportion of bacterial isolates with patients’ age and sex. P value of <0.05 was considered to indicate statistically significant differences.

## Results

A total of 575 patients specimens were collected and submitted for culture and drug susceptibility tests from patients with clinical evidence of infections from different sites. The subjects included 349 (60.7%) females and 226 (39.3%) males. One hundred fifty-three (26.6%) were in the age group of ≤18 years. Overall, 280 (48.7%) of infections had aerobic bacterial isolates. The proportion of bacterial infection was 116 (51.3%) in males and 164 (47.6%) in females (Table 1). Wound 238 (41.4%) and urine 108 (18.8%) samples were the most frequent specimens processed (Table 2).

**Table 1:** Age and sex distribution of common bacterial isolates from various site of infections among patients at Debre Markos Referral Hospital (2011–2014)

Type of isolate	Sex			Age in years							
	Male (n = 226)	Female (n = 349)	P value	0–4 (n = 50)	5–9 (n = 31)	10–14 (n = 43)	15–18 (n = 29)	19–30 (n = 262)	31–44 (n = 84)	45–90 (n = 76)	P value
	N (%)	N (%)		N (%)	N (%)	N (%)	N (%)	N (%)	N (%)	N (%)	
*S. aureus*	38 (16.8)	62 (17.8)	0.76	3 (6)	1 (3.2)	10 (23.3)	5 (17.2)	46 (17.6)	23 (27.4)	12 (15.8)	0.02
*E. coli*	13 (5.8)	26 (7.4)	0.43	3 (6)	3 (9.7)	–	1 (3.4)	26 (9.9)	2 (2.4)	4 (5.3)	0.06
*P. aeruginosa*	12 (5.3)	18 (5.2)	0.94	3 (6)	4 (12.9)	1 (2.3)	1 (3.4)	13 (5)	1 (1.2)	7 (9.2)	0.11
*Salmonella* spp.	18 (8)	7 (2)	0.001	12 (24)	3 (9.4)	–	–	5 (1.9)	2 (2.4)	3 (3.9)	<0.001
*Shigella* spp.	8 (3.5)	2 (0.6)	0.008	4 (8)	2 (6.5)	1 (2.3)	–	1 (0.4)	2 (2.4)	–	0.001
*N. gonorrhoeae*	4 (1.8)	12 (3.4)	0.23	–	–	1 (2.3)	2 (6.9)	13 (5)	–	–	0.04
*S. pyogens*	8 (3.5)	9 (2.6)	0.51	1 (2)	5 (16.1)	8 (18.6)	–	3 (1.1)	–	–	<0.001
*S. pneumoniae*	8 (3.5)	8 (2.3)	0.24	–	2 (6.5)	3 (7)	3 (6.4)	2 (0.8)	6 (7.1)	1 (1.3)	0.002
*Klebsiella* spp.	5 (2.2)	6 (1.7)	0.68	–	–	–	–	10 (3.8)	1 (1.2)	–	0.14
Proteus spp.	1 (0.4)	3 (0.9)	NA	–	–	–	–	2 (0.8)	–	2 (2.6)	NA
*Citrobacter* spp.	1 (0.4)	2 (0.6)	NA	–	–	–	–	1 (0.4)	–	2 (2.6)	NA
*Enterobacter* spp.	–	3 (0.9)	NA	–	–	–	–	3 (1.1)	–	–	NA
*N. meningitidis*	–	3 (0.9)	NA	–	–	–	–	–	1 (1.2)	2 (2.6)	NA
*S. agalactiae*	–	3 (0.9)	NA	–	–	–	–	3 (1.1)	–	–	NA
NG	110 (48.7)	185 (53.2)	0.26	24 (48)	11 (35.5)	19 (44.2)	17 (58.6)	134 (51.1)	46 (54.8)	44 (57.9)	0.26

*NA* not applicable, *NG* no growth of organisms, *n* total number of patients in each category of sex and age

**Table 2:** Isolation rates of bacteria in clinical samples collected from different site of infections occurred among patients at Debre Markos Referral Hospital (2011–2014)

Type of isolated organism	Type of clinical specimens								
	Wound (n = 238)	Ear (n = 58)	Urine (n = 108)	Stool (n = 58)	Blood (n = 41)	Urethral discharge (n = 28)	Nasal/throat swab (n = 12)	CSF (n = 32)	Total (n = 575)
*S. aureus*	70 (77.8)	18 (32.7)	3 (6.3)	–	2 (10.5)	4 (19)	3 (33.3)	–	100 (35.4)
*E. coli*	5 (5.6)	2 (3.6)	27 (60)	1 (2.9)	4 (21.1)	–	–	0	39 (13.9)
*P. aeruginosa*	6 (6.7)	19 (34.5)	5 (10.4)	0	0	–	–	–	30 (10.7)
*Salmonella* spp.	–	–	–	24 (68.6)	1 (5.3)	–	–	–	25 (8.9)
*Shigella* spp.	–	–	–	10 (28.6)	–	–	–	–	10 (3.6)
*N. gonorrhoeae*	–	–	–	–	0	16 (76.2)	0	0	16 (5.7)
*S. pneumoniae*	0	6 (10.9)	–	–	7 (36.8)	–	2 (22.2)	1	16 (5.7)
*S. pyogenes*	3 (3.3)	7 (12.7)	0	–	3 (15.8)	–	4 (44.4)	0	17 (6.1)
*Klebsiella* spp.	0	4 (7.3)	7 (14.6)	0	0	–	–	–	11 (3.9)
*N. meningitidis*	–	–	–	–	2 (10.5)	–	0	1	3 (1.1)
*Proteus* spp.	3 (3.3)	0	1 (2.1)	–	0	–	–	–	4 (1.4)
*S. agalactiae*	–	–	2 (4.2)	–	0	1 (4.8)	0	–	3 (1.1)
*Enterobacter*	3 (3.3)	0	0	–	–	–	–	–	3 (1.1)
*Citrobacter*	0	0	3 (6.3)	–	0	–	–	–	3 (1.1)
Total isolation rate	90 (32.1)	56 (20)	48 (17.1)	35 (12.5)	19 (6.8)	21 (7.5)	9 (3.2)	2 (0.7)	280 (100)

*n* total number of clinical specimens processed to isolate pathogens

### Bacteria profile

From 280 aerobic bacteria isolates, 270 (96.4%) had single isolates while 10 (3.6%) had mixed ones (data not shown). Two (6.9%) *Neisseria gonorrhoeae* (*N. gonorrhoeae*) were isolated from patients aged 15–18 years (P = 0.04). On the other hand, *Salmonella* spp. (P < 0.001) and *Shigella* spp. (P = 0.001) were the most common isolates in stools in children less than 5 years of age (Table 1). Gram negative bacteria accounted for 146 (52.1%) of the isolates. As shown in Table 2, *S. aureus* 100 (35.4%) was the predominant isolate, followed by *E. coli* 39 (13.9%), *P. aeruginosa* 30 (10.7%) and *Salmonella* spp. 25 (8.9%). Furthermore: *S. aureus* 71 (78%) was the leading isolate followed by *P. aeruginosa* 6 (6.6%) and *E. coli 5* (5.5%) in wound infection. *P. aeruginosa* 19 (34.5%) was the predominant isolate followed by *S. aureus* 18 (32.7%) in ear infection. *E. coli* 27 (60%) was the leading isolate followed by *Klebsiella* spp. 7 (14.6%) in urinary tract infection. *S. pneumoniae* 7 (36.8%) was the most frequent isolate followed by *E. coli* 4 (21.1%) in blood stream infection.

### Antimicrobial susceptibility profiles of bacterial isolates

#### Gram positive bacteria

Overall Gram positive isolates were resistant to cotrimoxazole (80%), penicillin (89.7%), clindamycin (93.2%), and erythromycin (90.9%). Moreover, higher (>83%) resistance was exhibited to gentamicin, ampicillin and amoxicillin (Table 3). *S. aureus* isolates were resistant to cotrimoxazole (81.3%), ampicillin (85.4%), gentamicin (86.8%), amoxicillin (87.5%), erythromycin (96.8%), penicillin (93.8%) and clindamycin (93.2%). However, *S. aureus* isolates were susceptible to ceftriaxone and norfloxacin with resistance rates of 13.5–22.2% (Table 3).

**Table 3:** Antimicrobial resistance profiles of Gram positive bacteria isolates from various site of infections among patients at Debre Markos Referral Hospital (2011–2014)

Antimicrobials	*S. aureus*		*S. pyogenes*		*S. pneumoniae*		Total	
	# T	R%	# T	R%	# T	R%	# T	R%
Ampicillin	89	76 (85.4)	10	8 (80)	11	10 (90.9)	110	94 (85.5)
Gentamicin	68	59 (86.8)	3	0	ND	ND	71	59 (83.1)
Ceftriaxone	37	5 (13.5)	12	1 (8.3)	12	6 (50)	61	12 (19.7)
Norfloxacin	18	4 (22.2)	3	0	1	0	22	4 (18.2)
Ciprofloxacin	13	6 (46.2)	4	0	3	1 (33.3)	20	7 (35)
Chloramphenicol	27	9 (33.3)	8	5 (62.5)	1	0	36	14 (38.9)
Cotrimoxazole	16	13 (81.3)	3	3 (100)	1	0	20	16 (80)
Amoxicillin	72	63 (87.5)	2	0	ND	ND	74	63 (85.1)
Tetracycline	82	57 (69.5)	5	1 (20)	1	0	88	58 (65.9)
Erythromycin	62	60 (96.8)	3	0	1	0	66	60 (90.9)
Oxacillin	62	27 (43.5)	2	0	ND	ND	64	27 (42.2)
Penicillin	65	61 (93.8)	3	0	ND	ND	68	61 (89.7)
Clindamycin	59	55 (93.2)	ND	ND	ND	ND	59	55 (93.2)
Cloxacillin	4	2 (50)	1	0	1	0	6	2 (33.3)
Total	674	497 (72.6)	59	18 (30.5)	32	17 (54.4)	765	532 (71.2)

*# T* number of isolates tested against each antimicrobial agent, *R%* percent of isolates resistance to antimicrobial agent, *ND* not done

#### Gram negative bacteria

Majority of Gram negative isolates were resistant to cotrimoxazole (53.1%), amoxicillin (58.8%), ampicillin (70.4%) and gentamicin (76.9%). *Salmonella* spp. was resistant to cotrimoxazole (72.7%), amoxicillin (88.9%) and ampicillin (100%). Shigella isolates were 100% resistant to cotrimoxazole, norfloxacin and chloramphenicol. *N. gonorrhoeae* isolates were 100% resistant to norfloxacin, chloramphenicol, ciprofloxacin and tetracycline. Conversely, these isolates were susceptible to ceftriaxone (38.5%) (Table 4). *E. coli* isolates were resistant to gentamicin (69.6%) and tetracycline (75%). Moreover, *P. aeruginosa* isolates were resistant to tetracycline (57.1%) and gentamicin (87.5%). Details of drug resistance profiles of Gram negative bacteria are presented in Table 4.

**Table 4:** Antimicrobial resistance profiles of Gram negative bacteria isolates from various site of infections among patients at Debre Markos Referral Hospital (2011–2014)

Antimicrobials	*E. coli*		*P. aeruginosa*		*Salmonella* spp.		*Shigella* spp.		*N. gonorrhoeae*		Total	
	# T	%R	# T	%R	# T	%R	# T	%R	# T	%R	# T	%R
Ampicillin	21	7 (33.3)	20	13 (65)	22	22 (100)	8	8 (100)	ND	ND	71	50 (70.4)
Gentamycin	23	16 (69.6)	16	14 (87.5)	ND	ND	ND	ND	ND	ND	39	30 (76.9)
Ceftriaxone	30	1 (3.3)	25	0	20	9 (45)	6	3 (50)	13	5 (35.8)	94	18 (19.1)
Doxycycline	8	6 (75)	14	2 (14.3)	14	9 (64.3)	6	5 (83.3)	7	5 (71.4)	49	27 (55.1)
Norfloxacin	26	6 (23.1)	20	0	23	11 (47.8)	5	5 (100)	5	5 (100)	79	27 (34.2)
Ciprofloxacin	22	4 (18.2)	17	6 (35.3)	16	4 (25)	3	0	8	8 (100)	66	19 (28.8)
Chloramphenicol	23	3 (13)	13	3 (23.1)	18	4 (22.2)	6	6 (100)	5	5 (100)	65	21 (32.3)
Cotrimoxazole	9	1 (11.1)	8	4 (50)	11	8 (72.7)	4	4 (100)	ND	ND	32	17 (53.1)
Amoxicillin	4	1 (25)	3	1 (33.3)	9	8 (88.9)	ND	ND	1	0	17	10 (58.8)
Tetracycline	12	9 (75)	7	4 (57.1)	1	0	1	1 (100)	8	8 (100)	29	22 (75.9)
Erythromycin	1	0	4	2 (50)	2	2 (100)	1	1 (100)	ND	ND	8	5 (62.5)
Cloxacillin	2	0	2	0	3	0	1	1 (100)	1	0	9	0
Total	181	54 (29.8)	149	49 (32.9)	139	77 (55.4)	41	33 (80.5)	48	33 (68.8)	558	44.8

*# T* number of isolates tested against each antimicrobial agent, *R%* percent of isolates resistance to antimicrobial agent, *ND* not done

### Multidrug- resistance profiles of the isolates

Overall, 24 (8.6%) of the isolates were susceptible to drugs tested, whereas 256 (91.4%) were resistant to one and more antimicrobials tested. Multidrug-resistance to two and more drugs were found in 213 (76.1%) of the isolates at a time (Table 5). The overall MDR rate among Gram positive (2–7 antimicrobial types) and Gram negative bacteria (2–6 antimicrobial types) isolates were 113 (84.6%) and 96 (72.2%), respectively (Table 5). Species specific MDR rate is depicted in Table 5.

**Table 5:** Antibiogram of common pathogenic bacteria isolated from different sites of infections at Debre Markos referral Hospital (2011–2014)

Bacterial species	R0	R1	R2	R3	R4	R5	R6	R7	Over all, MDR
*S. aureus* (n = 100)	5 (5)	5 (5)	8 (7)	12 (12)	23 (23)	16 (16)	19 (17)	12 (12)	90 (90)
*E. coli* (n = 39)	6 (15.4)	9 (23.1)	10 (25.6)	7 (17.9)	3 (8.8)	2 (5.9)	2 (5.1)		24 (61.5)
*P. aeruginosa* (n = 30)	3 (10)	5 (16.7)	8 (26.7)	9 (30)	3 (10)	2 (6.7)	0	0	22 (73.3)
*Klebsiella* spp. (n = 11)	1 (9.1)	1 (9.1)	2 (18.2)	4 (36.4)	3 (27.3)	0	0	0	9 (81.8)
*S. pyogenes* (n = 17)	3 (17.6)	1 (5.9)	7 (41.2)	4 (23.5)	2 (11.8)	0	0	0	13 (76.5)
*S. pneumoniae* (n = 16)	0	8 (50)	5 (31.3)	1 (6.3)	2 (12.5)	0	0	0	8 (50)
*Salmonella* spp. (n = 25)	0	3 (12)	3 (12)	8 (32)	5 (20)	4 (16)	2 (8)	0	22 (88)
*Shigella* spp. (n = 10)	0	4 (40)	0	2 (20)	2 (20)	2 (20)	0	0	6 (60)
*N. gonorrhoeae* (n = 16)	2 (12.5)	4 (25)	5 (31.3)	2 (12.5)	3 (18.8)	0	0	0	10 (62.5)
*N. meningitidis* (n = 3)	0	0	2 (66.7)	1 (33.3)	0	0	0	0	3
*Proteus* spp. (N = 4)	1 (25)	1 (25)	1 (25)	1 (25)	0	0	0	0	2
*Citrobacter* spp. (N = 3)	1 (33.3)	1 (33.3)	1 (33.3)	0	0	0	0	0	1
*Enterobacter* spp. (N = 3)	1 (33.3)	0	1 (33.3)	0	0	0	1	0	2
*S. agalactiae* (N = 3)	1 (33.3)	1 (33.3)	0	0	0	1 (33.3)	(33.3)	0	1
Total (n = 280)	24 (8.6)	43 (15.4)	53 (18.9)	51 (18.2)	46 (16.4)	27 (9.6)	24 (8.6)	12 (4.3)	213 (76.1)

R0: susceptible to all antimicrobials tested; R1, R2, R3, R4, R5, R6, R7: Resistance to one, two, three, four, five, six and seven antimicrobials, respectively

## Discussion

Multidrug-resistant bacterial infection becomes a real threat in developing countries including Ethiopia. In the study area the majority of pathogenic bacteria isolated from various clinical specimens such as wound, urine, stool, ear and urethral discharge were drug resistant.

In the present study, Gram negative bacteria were the dominant isolates similar to previous studies in other areas of Ethiopia [14–17] and elsewhere [18]. However, *S. aureus* was the most frequent isolate followed by *E. coli*, *P. aeruginosa* and *Salmonella* spp. This trend agrees with reports of other studies in Ethiopia [14–17]. The possible reason for the high frequency is that majority of these isolates are normal flora on skin and gut of healthy individuals. When they get breach on skins and soft tissues and displaced from their resident to other sterile sites they can easily disseminate. Moreover, most of these bacteria are commonly found in the hospital environment which might increase the proportion of wound, ear and urinary tract infection and cross contamination among admitted patients.

The high proportion of *S. aureus* followed by *P. aeruginosa* in wound infection in this study might be because of endogenous source of infection or contamination from the environment such as contamination of surgical instruments with the disruption of natural skin barrier as these bacteria are a common bacterium on surfaces, easily finds their way into wounds [16]. Moreover, the high frequency of *P. aeruginosa* in ear infection could be related to the ability of *P. aeruginosa* to survive in competition with other organisms and resist antibiotics.

In this study *N. gonorrhoeae* was most frequently isolated in patients with the age groups of 15–18 years compared to other age groups. This is in agreement with a study done in other part of Ethiopia [19]. This might be because individuals at this age are key populations of higher risk for sexually transmitted infections like gonorrhea acquisition or transmission. Hence between these ages young individuals undergo transition in life style, maturity and legal rights which will place them at different vulnerabilities at different time points.

*Salmonella* and *Shigella* spp. were the most common isolates in stool in children less than 5 years of age, which is in line with studies conducted in Ethiopia [20] and China [21]. It is true that children with in this age group are more susceptible to shigellosis and salmonellosis primarily because of lack of resistance, previous exposure to infections, poor personal hygiene and higher exposure to contaminated environments.

In this study, overall high levels of resistance were demonstrated against amoxicillin, clindamycin, erythromycin and penicillin. These were consistent with resistance rates obtained from previous studies in Ethiopia [6, 16] and India [22]. However, majority of bacteria isolates revealed lower levels of resistance against ciprofloxacin, ceftriaxone, norfloxacin and chloramphenicol. Moreover, Gram positive bacteria showed high levels of resistance (82.1–98.5%) to ampicillin, gentamicin, cotrimoxazole, amoxicillin, penicillin and clindamycin. This finding is similar with studies carried out in Ethiopia [23] and India [22] where 75–100% resistance to the above antibiotics reported. Similarly, *S. aureus* isolates revealed high levels of resistance (81.3–96.8%) to the above mentioned antibiotics. These results are in agreement with the reports from Ethiopia and other countries [6–9, 17, 22].

In the present study, *N. gonorrhoeae* isolates revealed 100% resistant to ciprofloxacin, norfloxacin and tetracycline. This agrees with studies carried out in Ethiopia [19, 24], Uganda [25], Port Elizabeth [26] and Iran [27]. This might be because of easy availability, over and indiscriminate use of these drugs outside the hospitals. In contrast, *N. gonorrhoeae* exhibited low level of resistance to ceftriaxone. This was coherent with reports of other studies elsewhere [19, 24, 28, 29].

In the present study Salmonella spp. showed high levels of resistance (85.7–100%) against amoxicillin and ampicillin. These were consistent with previous studies done in Ethiopia [6, 20, 30] and Madagascar [31]. In the current study, it was found that *Shigella* spp. revealed high level of resistance to cotrimoxazole (100%). This was in agreement with reports from other studies [6, 31–34]. This might be due to the indiscriminate drug prescription by clinicians in the study area since culture and susceptibility testing were not employed in the previous years.

In the present study, *E. coli* was the most frequently isolated bacteria in urinary tract infection and a common isolate in bacteremia. This shows that urosepsis is a major cause of infection. Majority of *E. coli* resisted the antimicrobials gentamicin and tetracycline. A similar result was documented from other studies [16, 35].

In the case of *P. aeruginosa*, high level of resistance (87.5%) to gentamicin and considerable level of resistance (57%) to tetracycline were recorded. This was consistent with studies conducted in other parts of Ethiopia [7, 15]. However, the rate of resistance of *P. aeruginosa* against gentamicin is different from other studies [9, 15, 16]. On the other hand, *P. aeruginosa* was 100% susceptible to ceftriaxone and norfloxacin. A similar result was documented from other studies [8, 14].

The overall MDR (two and above drugs tested) of the isolates in this study was 76.1% which was coherent with studies conducted in other parts of Ethiopia [3, 14] where 63.3–85% MDR rate was reported. In this study, 85% of Gram positive bacteria (*S. aureus*, *S. pyogenes* and *S. pneumoniae*) demonstrated MDR. This was similar with the 77 and 65.2% MDR rate documented for these bacteria in Ethiopia [23, 35]. However, it was lower than 98.6 and 100% MDR reported in other places of Ethiopia [7, 36]. The possible explanation for such disparity might be difference in type of organism isolated, study population, antimicrobial prescription pattern, study area in terms of laboratory infrastructure, infection prevention practices and up to-date knowledge of clinicians on AMR [3]. About 90% of *S. aureus* also became MDR of which 12% were resisted to seven drugs tested.

The overall MDR rate of Gram negative bacteria tested for seven types of drugs was 68.5% in this study. This finding is higher than studies conducted in other parts of Ethiopia where 51–59.3% MDR Gram negative bacterial isolates from different types of infections were reported [23, 32]. This might be due to the drugs having been in use for much longer time.

Concerning species specific MDR profiles, *Salmonella* and *Shigella* spp. showed MDR, 88 and 60%, respectively. This was consistent with previous studies done in Ethiopia [6, 20, 30, 32, 33] and Madagascar [31]. It was also found that 61.5% of *E. coli* was MDR. A similar result was documented from other studies [16, 35]. However, Godebu et al. documented 23.3% rate of MDR *E. coli* in Ethiopia. In the case of *P. aeruginosa*, 75.9% was MDR. This was consistent with studies conducted in Ethiopia [7, 15] where 100% MDR rate reported. Lastly, 62.5% of gonococci isolates in the present study revealed multidrug-resistance that needs further large scale study on antibiogram of *N. gonorrhoeae* to control the alarming spread of *N. gonorrhoeae* and other pathogenic species.

Because of retrospective nature of the study, detail information on patient profiles could not be obtained. For some of the pathogen and antibiotic combination, numbers tested were small and this limits the interpretation of the data. Furthermore, anaerobic bacteria were not isolated due to limited laboratory infrastructure.

## Conclusions

This study revealed that *S. aureus, E. coli, P. aeruginosa, Salmonella* spp. and *N. gonorrhoeae* were the most common isolates in clinical samples. Isolates showed high levels of resistance to ampicillin, gentamicin, cotrimoxazole, amoxicillin, penicillin and clindamycin. However, lower levels of resistance were showed for ciprofloxacin, ceftriaxone, norfloxacin and chloramphenicol. Majority of Gram positive and Gram negative isolates showed MDR. Therefore, treatment of common bacterial infections in the study area needs to be guided by antibiotic susceptibility testings.

## Abbreviations

AMR: antimicrobial resistance
ATCC: American Type Culture Collection
CLBI: Clinical and Laboratory standards Institute
CSF: cerebrospinal fluid
DMRH: Debre Markos Referral Hospital
MDR: multidrug-resistant
